# Cuckoo eyes are an important identification cue for the Oriental reed warbler host

**DOI:** 10.1016/j.ijppaw.2025.101038

**Published:** 2025-01-10

**Authors:** Hanlin Yan, Longwu Wang, Laikun Ma, Wei Liang

**Affiliations:** a Ministry of Education Key Laboratory for Ecology of Tropical Islands, Key Laboratory of Tropical Animal and Plant Ecology of Hainan Province, College of Life Sciences, Hainan Normal University, Haikou 571158, China; b School of Life Sciences, Guizhou Normal University, Guiyang 550001, China; c Department of Biology and Food Science, Hebei Normal University for Nationalities, Chengde 067000, China

**Keywords:** 3D printed model, Antiparasitic behavior, Common cuckoo, Identification cue, Parasite recognition

## Abstract

Successful recognition of parasites through effective identification cues can reduce the cost of anti-parasitic defenses by the host. Features on the front of the body such as the eyes may be important clues for the prey to perceive and recognize the parasite. In this study, we investigated whether the eyes of common cuckoos (*Cuculus canorus*), an obligate avian brood parasite, served as identification cues for its common host, the Oriental reed warbler (*Acrocephalus orientalis*). We displayed dummies of 3D printed common cuckoos and Oriental turtle doves (*Streptopelia orientalis*) with their eyes covered or not-covered near the nests of breeding Oriental reed warblers to test effect of the eyes on defense behaviors of the warblers towards these dummies. Oriental reed warblers significantly reduced attacks on the common cuckoos with eyes covered compared to those with eyes not-covered. However, there was no significant difference between the attacks on the Oriental turtle doves with not-covered eyes and those with covered eyes. Our results suggest that Oriental reed warblers use cuckoo eyes as an important discrimination cue. We explored for the first time the use of the cuckoo's eye as an important discriminative cue by Oriental reed warblers via visual manipulation of 3D printed cuckoos, which provides a new experimental validation of the host Oriental reed warbler's anti-parasite strategy in terms of visual cues. However, Future research should consider testing more cuckoo species and their hosts and further validating the identification cues of the eyes and other body parts in additional geographic populations.

## Highlights

• Successful recognition of parasites through effective identification cues can reduce the cost of anti-parasitic defenses by the host.
• Oriental reed warblers (*Acrocephalus orientalis*) host reduced attacks on common cuckoos (*Cuculus canorus*) with eyes covered.
• Oriental reed warblers use cuckoo eyes as an important discrimination cue.

## Graphical abstract

Oriental reed warblers (*Acrocephalus orientalis*) host significantly reduced attacks on common cuckoos (*Cuculus canorus*) with eyes covered compared to those with eyes not-covered, while there was no difference between the attacks on Oriental turtle doves (*Streptopelia orientalis*) with not-covered eyes and those with covered eyes, suggesting that Oriental reed warblers use cuckoo eyes as an important discrimination cue.

**Figure:**
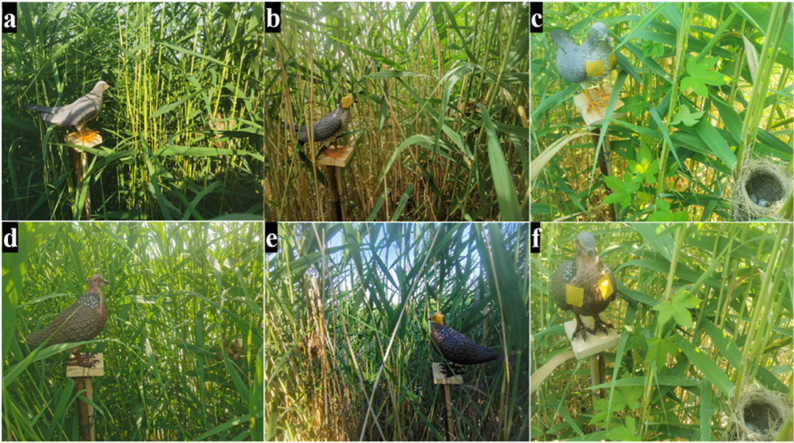

## 1 Introduction

Direct conflict and deceptive antagonisms were involved during the interactions between avian brood parasites and their hosts (Davies, 2000; Feeney et al., 2012). The nest defense of hosts has been proven effective in preventing parasites from laying eggs (Welbergen and Davies, 2009; Feeney and Langmore, 2013). However, to defense effectively, hosts should be able to recognize parasites and regard them as unique threats (Welbergen and Davies, 2008; Campobello and Sealy, 2010; Davies and Welbergen, 2009). It has been found that some host species can distinguish between different parasites (Trnka et al., 2012; Trnka and Prokop, 2012; Yang et al., 2014). Although it is unclear how various hosts recognize their parasites, it is likely to involve some morphological features of the parasite (e.g., head and beak), and possibly also in combination with certain features (e.g., the yellow eye rings and barring on the underside of the body: Grim, 2005; Trnka et al., 2012). Each of these features may have some degree of importance (Welbergen and Davies, 2011; Trnka and Prokop, 2012; Nováková et al., 2017).

In addition, common cuckoos (*Cuculus canorus*) are both nest parasites and nest predators, i.e., common cuckoos display destructive or predatory behaviors to increase their parasitism chances (Šulc et al., 2020). This forces their hosts to nest and reproduce again (Zahavi, 1979; Swan et al., 2015). One of the cues the prey used to determine predation risk is presence of predator's eyes or eye gaze where it is looking (Kyle, 2021). For example, Emery (2000) showed that eye gaze is considered a cue indicating threat in social situations or predator-prey interactions. Additionally, when the eyes of avian predators are present, domestic chickens (*Gallus gallus domesticus*) exhibit stronger anti-predator behaviors compared to when the eyes are removed (Scaife, 1976). Based on the results of previous studies, it appears that hosts react differently to nest parasites than to other non-threatening invaders, suggesting that hosts can recognize brood parasites as distinct enemies (Sealy et al., 1998; Grim et al., 2011; Campobello and Sealy, 2010; Røskaft et al., 2002). However, only a few studies have explored the specific cues hosts use to identify the parasite (Gill et al., 1997; Davies and Welbergen, 2008; Welbergen and Davies, 2011; Trnka et al., 2012).

Oriental reed warblers (*Acrocephalus orientalis*) are one of the most common hosts of common cuckoos, reaching a high degree of coevolutionary adaptation (Li et al., 2016; Ma et al., 2018; Ma and Liang, 2021; Wang et al., 2022). Previous studies have shown that Oriental reed warblers not only have strong egg discrimination abilities (Ma and Liang, 2021; Ma et al., 2024), but also strong nest defense (Wang et al., 2023). Oriental reed warblers exhibit a certain level of defensive aggression towards common cuckoos present near their nests during the breeding season (Li et al., 2016; Ma and Liang, 2021; Trnka et al., 2023). In visually, the Oriental reed warblers are able to distinguish between common cuckoos and harmless oriental turtle doves, and they are more likely to mob cuckoos than sparrowhawk (*Accipiter nisus*) (Ma et al., 2018). In recent years, there have also been many reports on the active defense mechanisms of the host and their impact on parasite adaptation (Dillenseger, 2024). However, how Oriental reed warblers recognize their parasites, the common cuckoos, is still not well understood. In this study, we displayed different types of cuckoo dummies (with eyes covered or eyes not-covered) near the nests of Oriental reed warblers to observe the nest defense behaviors of the warblers toward these dummies. The aim was to explore whether the eyes of common cuckoos serve as recognition cues for the host Oriental reed warblers against the parasites. During the breeding season, if parasites appear near the nest, the yellow eyes on the cuckoos' bodies will be clearly visible from the typical direction of approach by the host (from above), while the striped lower part may be difficult to see unless approaching the cuckoo at the height of the nest (visually obscured by the upper portion) (Trnka et al., 2012). The Oriental reed warbler was considered just a subspecies of the great reed warbler (*Acrocephalus arundinaceus*) until recently (Zheng, 2023). That great reed warbler uses yellow eyes as a the most important discrimination cue is well tested and known (Trnka et al., 2012). Given that there might be population differences and effects of different brood parasite community in Asia, further experiments for Oriental reed warblers are necessary. We predicted that Oriental reed warblers would be less likely to attack eye-covered dummies, meaning that the eyes of common cuckoos may be one of the most important cues used by Oriental reed warblers to identify parasites.

## 2 Materials and methods

### 2.1 Study area and study species

Sifangtuozi Farm (46°00′-22′ N, 123°46′-57′ E) is located in Jilin, northeast China. There are large numbers of reeds growing in areas such as farm ridges, diversions, ditches, and ponds around the farm. These areas have become breeding grounds for the Oriental reed warbler (Trnka et al., 2023).

Oriental reed warblers primarily rely on reed habitats for breeding and are summer residents in the study area. The breeding season occurs from late May to early August each year (Yang et al., 2016, 2017). They were identified as the main hosts of common cuckoos in the study area and exhibited strong aggressive behavior towards them (Li et al., 2015; Yu et al., 2019). We conducted field experiments for this study during the breeding season of year 2022.

### 2.2 Experimental design

It has been demonstrated that the effects of 3D printed animal models do not differ from the experimental effects of bird specimens in Oriental reed warblers (Chen et al., 2022). Therefore, our experiments used dummies of common cuckoos (referred to as cuckoo) and Oriental turtle doves (*Streptopelia orientalis*) (referred to as dove), both of which are 3D printed models (also see Trnka et al., 2023). To avoid pseudo-replication, two individuals of each dummy type were prepared in advance, and one of them was randomly selected for each experiment (e.g., Welbergen and Davies, 2009).

We categorized the responses of Oriental reed warblers into two types for non-aggressive behavior and aggressive behavior. The non-aggressive behavior included (1) No response: A common cuckoo was not detected or quietly observed by the Oriental reed warbler without any apparent reaction and (2) Alert: The Oriental reed warbler approached the nest (hopping onto a reed stem) but issued an alert call from a safe distance (more than 1 m away). The aggressive behavior included (3) Mobbing: The Oriental reed warbler exhibited jumping, flying, or rioting around/above the dummy, continuously emitting alarm calls and distress signals and (4) Attack: The Oriental reed warbler directly attacked the dummy, resulting in physical contact (see also Li et al., 2015; Ma et al., 2018; Chen et al., 2022; Trnka et al., 2023).

**Fig. 1:**
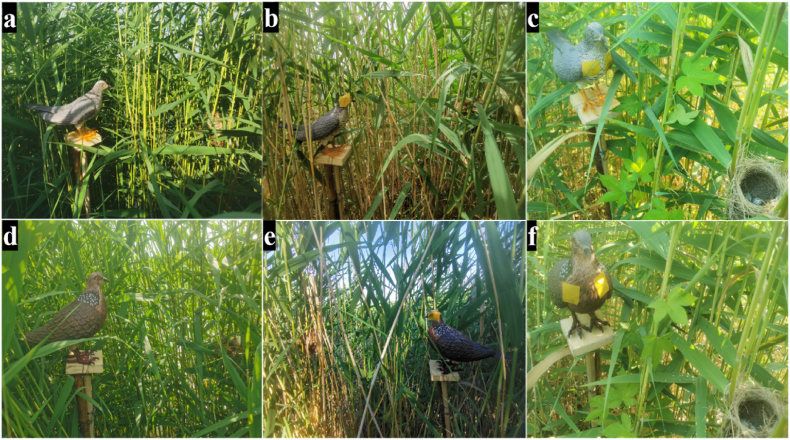
Displaying dummy 0.5 m from the side of Oriental reed warbler's nest, with both of dummy's heads facing the nest to observe Oriental reed warbler's reaction to seeing dummy when it returns to the nest (the letter a refers to the “natural” 3D printed cuckoo, b refers to the covered 3D printed cuckoo, c refers to the not-covered 3D printed cuckoo, d refers to the “natural” 3D printed dove, e refers to the covered 3D printed dove and f refers to the not-covered 3D printed dove).

**Fig. 2:**
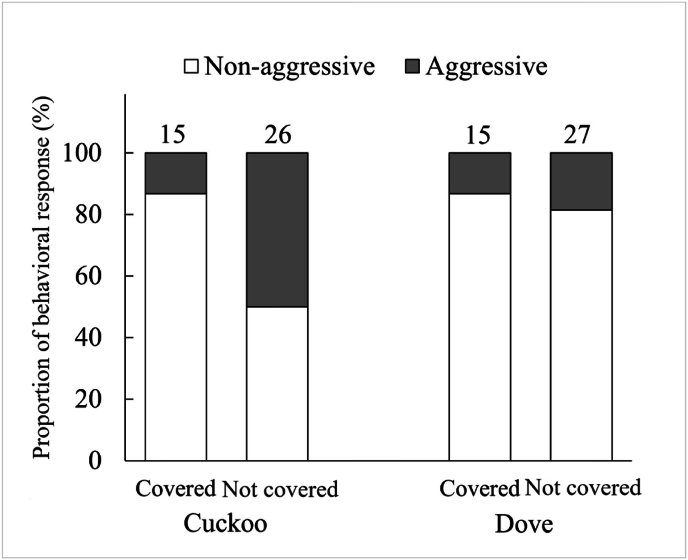
Behavioral responses of Oriental reed warblers to different types of 3D printed dummies (the number at the top of each column is the total sample size, and the percentage for each region of the column represents the percentage of the sample size of the reaction type in that region to the total sample size).

Based on the eye types, the field dummy experiments were divided into three groups including (1) “natural” 3D printed group: intact “natural” 3D printed dummies with no manipulation (Fig. 1a and d), (2) eyes covered group: non-transparent adhesive tape applied to the eyes of both types of dummies, covering the eyes (Fig. 1b and e), and (3) eyes not-covered group: same adhesive yellow tape applied to both types of dummies, covering the chest but not the eyes to eliminate the effects of tape itself (Fig. 1c and f). Each experiment group included two trials which used one of the two types of cuckoo and dove dummies.

During the experiment, the dummy randomly selected were first fixed on a prepared display stand (in a standing position). We chose nests with more than three eggs for experimental testing. The dummy and display stand were placed 0.5 m in front of the focal nest of the Oriental reed warbler (random locations around the focal nest), with the head of the dummy facing the nest. Next, a video recorder was placed beside the dummy and the nest for recording (Ou Chuang A8; Xiamen Shangyu Huajin Electronic Technology Co., Ltd. Xiamen, China). The responses of the Oriental reed warblers upon seeing different types of dummies displayed at the nest site were documented based on observations by the experimenters and recordings from the video recorder. When the parent birds approached the nest and discovered the dummies, the experiment began and lasted for 5 min. If the parents were not observed near the nest within 15 min after the dummy was exposed, the experiment was stopped, and the data from that trial were excluded from the analysis (Trnka et al., 2023). Only one experiment group including two type of dummies was tested for a focal breeding nest. After first dummy type in the same group, there was a minimum interval of 1 h before the next trial to minimize the possibility of habituation or sustained aggression (e.g., Ma et al., 2018).

### 2.3 Statistical analyses

Statistical analysis was performed using IBM SPSS Statistics 20 (IBM Inc., Armonk, NY, USA) for Windows, employing Fisher's exact test and Chi-square test for comparisons between different probabilities for different behavior type of Oriental reed warblers. All tests were two-tailed with a significance level set at *P* < 0.05, and data are presented as mean ± standard deviation (Mean ± SD).

### 2.4 Ethical note

The study was conducted in compliance with the law of China. Experimental procedures in China were in accordance with the Animal Research Ethics Committee of Hainan Provincial Education Centre for Ecology and Environment, Hainan Normal University (no. HNECEE-2012-003) and Guizhou Normal University (No. GZNUECEE-2021-001).

## 3 Results

For the “natural” cuckoo dummies in experiment group (1), the aggressive behavior was observed in a total of 53% of nests and no aggression in 47% nests in the Oriental reed warbler host. For the covered cuckoo dummies in experiment group (2), the aggressive behavior was observed in 13% nests and no aggression in 87% nests. For the not-covered cuckoo dummies in experiment group (3), the aggressive behavior was observed in 44% nests and no aggression in 56% nests (Table 1).

**Table 1:** Behavioral responses of Oriental reed warblers to different 3D printed dummy types.

Dummy type	Eye type	Response	
		Non-aggressive behavior	Aggressive behavior
Common cuckoo	“natural” group (n = 17)	8	9
	eye not-covered group (n = 9)	5	4
	eye covered group (n = 15)	13	2
Oriental turtle dove	“natural” group (n = 18)	14	4
	eye not-covered group (n = 9)	8	1
	eye covered group (n = 15)	13	2

Similarly, for “natural” dove dummies in experiment group (1), the aggressive behavior was observed in 22% nests and no aggression in 78% nests in the Oriental reed warbler host. For the covered dove dummies in experiment group (2), the aggressive behavior was observed in 13% nests and no aggression in 87% nests. For not-covered dove dummies in experiment group (3), only 11% nest was observed aggressive behavior among the total 9 nests (Table 1).

The behavioral response of Oriental reed warblers to the “natural” and eye not-covered groups was similar (Fisher's exact test, for cuckoo dummies, *P* = 1.000, and for dove dummies, *P* = 0.636); therefore, the two groups of data were combined to calculate the eye not-covered group. The response of Oriental reed warblers to cuckoo dummies in the eye not-covered group was significantly stronger than that of the eye covered group (Chi-square test, ꭓ^2^ = 5.512, *P* = 0.019) (Fig. 2), while the response to dove dummies in the eye not-covered group was similar to that of the eye covered group (Fisher's exact test, *P* = 1.000). Additionally, within the eye not-covered group, the response of Oriental reed warblers to cuckoo dummies was significantly stronger than that to dove dummies (Chi-square test, ꭓ^2^ = 5.853, *P* = 0.016), whereas in the eye not covered group, there was no significant difference in the warbler's response to the two types of dummies (Fisher's exact test, *P* = 1.000).

## 4 Discussion

Our study indicated that Oriental reed warblers exhibited fewer attacks toward cuckoo dummies with covered eyes compared to those with not-covered eyes. Furthermore, Oriental reed warblers exhibited significantly stronger aggressive behavior toward cuckoo dummy compared to dove dummy when the eyes of the dummy were not covered, and the significantly difference disappeared after the eyes were covered.

Previous studies have showed that the host exhibits different defensive behaviors against cuckoos near the nest than other species due to the high cost of nest cuckoo parasitism (Welbergen and Davies, 2008, 2009; Trnka et al., 2012; Li et al., 2015; Ma et al., 2018). Some aggressive strikes may be also fatal to cuckoos (e.g. Šulc et al., 2020; Zhao et al., 2022). Similar results were found in our study. Oriental reed warblers exhibit higher levels of aggression toward cuckoo dummy compared to dove dummy, indicating that they considered the cuckoo to be a threatened species. This may be because the Oriental reed warblers is the main host of the common cuckoo in this region, and the two species have a long history of coevolution and interaction, which the Oriental reed warblers have evolved excellent recognition ability to identify the cuckoo as unique threats (Li et al., 2015; Ma et al., 2018; but see Trnka et al., 2023).

However, this cognitive and response difference significantly disappeared after the eyes of cuckoo dummy were covered. Additionally, when responding to common cuckoo dummies with covered versus not-covered ones, Oriental reed warblers significantly reduce their aggression toward the cuckoo dummies with adhesive tape applied to the eyes. These results suggest that eyes of cuckoo dummy play an important role in the risk recognition of Oriental reed warblers.

Previous studies have shown that birds anticipate risk based on eye cues from enemies appearing around them (Carter et al., 2008; Lee et al., 2013; Goumas et al., 2019). For example, Davidson et al. (2015) suggest that jackdaws (*Corvus monedula*) may integrate predator identity and gaze cues to make defensive responses, thus avoiding potential threats. Similar result was also found in Kyle (2021), who demonstrated that two species of tits, Carolina chickadees (*Poecile carolinensis*), and tufted titmice (*Baeolophus bicolor*) can utilize the presence of a predator's eyes (covered versus not-covered) to determine predation risk. In the research of avian brood parasitism, Trnka et al. (2012) found that great reed warblers (*Acrocephalus arundinaceus*) focused more on cues located in the anterior region of the intruder's body and primarily rely on a single identification cue, namely the color of the intruder's iris and eye ring. Our findings are similar to the above results, suggesting that the eyes of the common cuckoo may serve as one of the identification cues for Oriental reed warblers to recognize brood parasites. This may be because the relative importance of the parasite's eyes compared to the phenotype of the lower body may reflect the ecological context of the interaction between the host and the parasite. For example, when approaching from the regular direction (from above), the yellow eyes of the cuckoo are prominent on its body, while the striped lower part is difficult to see (obscured by the upper visual field; Trnka et al., 2012).

Birds will most strongly avoid a pair of iris-bright “stalking, staring eyes” (Leisler and Schulze-Hagen, 2013), and in terms of visual recognition cues, the iris background coloration of the common cuckoo is a mimicry of the characteristics of *Accipiter* hawks to facilitate access to host nests (Trnka et al., 2012; Thorogood and Davies, 2013). The yellow iris and orbits of the common cuckoo enhance the so-called “penetrating gaze”, making the common cuckoo's eye not only striking but also emphasizing its pointing direction (Leisler and Schulze-Hagen, 2013). This is more conducive to the common cuckoo's ability to intimidate its hosts, causing them to lower their own nest defenses, thus favoring the cuckoo's parasitism (Trnka et al., 2012; Leisler and Schulze-Hagen, 2013), thereby reducing host reproductive success and causing severe reproductive losses to the host. It is also an adaptive strategy planned by the parasite in concert with the host. Thus, the results of our experimental tests may indirectly validate the study of the common cuckoo eyes as one of the main recognition cues for the host Oriental reed warblers.

In addition, the Oriental reed warblers may use other co-varying features (e.g., head shape or general posture) as identification cues, because of similarities in size, flight patterns, and plumage between the cuckoo (*Cuculus* spp.) (Cuculinae) and the sparrowhawk (genus *Accipiter*) (Davies and Welbergen, 2008). Previous studies have suggested that this is as visually beneficial to the host as the “eye” cues, because similarity to predators aids parasitism, on the one hand it can be more effective in fending off small birds that hate it, but conversely it can also stimulate aggression in host birds so that parasites can better estimate the density or frequency of potential hosts in an area (Welbergen and Davies, 2012; Trnka et al., 2012; Leisler and Schulze-Hagen, 2013). In addition, the introduction of the yellow tape itself in our experiments could have introduced a potential bias, even though we set up experimental group 3 (eyes not-covered group) for eliminating the potential effect of the yellow tape on the recognition, our sample size was small, and thus this possibility should not be completely ruled out, which need to be addressed in future experiments.

In summary, our findings showed that the Oriental reed warbler was more reluctant to attack cuckoos with covered eyes, meaning that common cuckoos' eyes influenced the Oriental reed warblers' defensive attack behavior. This suggests that Oriental reed warblers may use the eyes of the common cuckoo as a primary identification cue (also see Trnka et al., 2012). However, given that we did not capture potential confounders in our field experiments and the sample size in our study was relatively small, we acknowledge this limitation of our study. Therefore, we suggest expanding sample sizes in future studies and conducting experimental tests on different geographic populations. In addition, future research should consider testing with more cuckoo species and their hosts and further validating the identification cues of the eyes and other body parts in cuckoos.

## CRediT authorship contribution statement

**Hanlin Yan:** Writing – original draft, Methodology, Investigation, Formal analysis, Data curation. **Longwu Wang:** Writing – review & editing, Supervision, Resources, Conceptualization. **Laikun Ma:** Validation, Methodology, Formal analysis. **Wei Liang:** Writing – review & editing, Validation, Supervision, Funding acquisition, Conceptualization.

## Data availability statement

Data used for this study was provided as supplementary material (Data Table S1).

## Ethical approval

The study was conducted in compliance with the law of China. Experimental procedures in China were in accordance with the Animal Research Ethics Committee of Hainan Provincial Education Centre for Ecology and Environment, Hainan Normal University (no. HNECEE-2012-003) and Guizhou Normal University (No. GZNUECEE-2021-001).

## Funding

This work was supported by the 10.13039/501100001809National Natural Science Foundation of China (Nos. 32270526 to WL and 32101242 to 10.13039/100006186LM).

## Declaration of interest

The authors declare that they have no known competing financial interests or personal relationships that could have appeared to influence the work reported in this paper.
